# Gender differences in the relationship between the systemic immune-inflammation index and all-cause and cardiovascular mortality among adults with hypertension: evidence from NHANES 1999-2018

**DOI:** 10.3389/fendo.2024.1436999

**Published:** 2024-10-08

**Authors:** Ting Cheng, Dongdong Yu, Qi Tang, Xingying Qiu, Geng Li, Li Zhou, Yue Yang, Zehuai Wen

**Affiliations:** ^1^ Second Clinical Medical College, Guangzhou University of Chinese Medicine, Guangzhou, China; ^2^ Department of Cardiovascular, First Affiliated Hospital of Anhui University of Chinese Medicine, Hefei, China; ^3^ Second Affiliated Hospital of Guangzhou University of Chinese Medicine (Guangdong Provincial Hospital of Chinese Medicine), Guangdong Provincial Academy of Chinese Medical Sciences, Guangzhou, China; ^4^ Science and Technology Innovation Center, Guangzhou University of Chinese Medicine, Guangzhou, China

**Keywords:** systemic immune-inflammation index, all-cause mortality, cardiovascular mortality, hypertension, gender differences

## Abstract

**Background:**

There are gender differences in hypertension and the effect of gender on the relationship between systemic immune-inflammation index (SII) and mortality in hypertensive patients is unclear.

**Methods:**

Hypertensive patients (n=7444) from ten cycles of the National Health and Nutrition Examination Survey (NHANES) spanning 1999 to 2018 were enrolled in this study. The maximally selected rank statistics method was employed to identify the optimal cut-off value for the SII. Survey-weighted Cox regression analysis was utilized to explore the links between SII and all-cause and cardiovascular mortality. Kaplan-Meier method and time-dependent receiver operating characteristic curve analysis was conducted to assess the predictive accuracy of SII for mortality.

**Results:**

Whether SII was considered as a numerical variable or as a binary variable (higher- and lower-SII groups), higher SII levels were associated with a higher risk of all-cause and cardiovascular mortality in female hypertensive patients (all *P* < 0.001), but no such association was observed in the males. The area under the curve of the SII was 0.602, 0.595, and 0.569 for 3-, 5-, and 10-year all-cause mortality, respectively, in females, but was 0.572, 0.548, and 0.554 in males. High SII levels interacted with the poverty income ratio and physical activity to affect mortality in the male population (*P* for interaction < 0.05), and there was an interaction between race and SII in the female cardiovascular mortality rate (*P* for interaction < 0.05).

**Conclusion:**

Higher levels of SII may be closely related to the high risk of all-cause and cardiovascular mortality in hypertensive patients, and the results showed that this relationship is more significant and stable in the female group. High SII interacts with PIR, physical activity, and race to affect the mortality rate in different gender populations.

## Introduction

In the past few decades, hypertension has been one of the major global public health issues. According to reports, in 2010, there were 1.39 billion people worldwide suffering from hypertension, accounting for 10% of the total global health care expenditure (1). Hypertension is an important modifiable risk factor for cardiovascular disease (CVD) and related target organ damage, and it is also a significant cause of increased risk for all-cause and cardiovascular mortality (2, 3). As such, it is essential to promptly recognize additional risk factors related to hypertension to prevent and slow its progression, mitigate associated target organ damage, and decrease the occurrence of adverse events.

Hypertension is a recognized major risk factor for CVD, which is the most common cause of death globally. Increasing evidence suggests that immune system dysregulation and inflammation are among the pathogenic mechanisms of hypertension. For example, a comprehensive study of genome-wide association studies (GWAS) has shown that some hypertension-related single nucleotide polymorphisms (SNPs) are directly or indirectly involved in the inflammatory process (4). A secondary analysis of the Canakinumab Anti-Inflammatory Thrombosis Outcomes Study (CANTOS) has indicated that specific anti-inflammatory treatments can somewhat reduce the occurrence of adverse cardiovascular events in hypertensive patients (5). In recent years, extensive attention has been paid to the relationship between white blood cell (WBC) count, WBC subtypes, and their combination indices with hypertension, cardiovascular disease risk, and all-cause and cardiovascular mortality (6, 7). The systemic immune-inflammation index (SII) is considered a novel biomarker based on circulating immune-inflammatory cells (neutrophils, platelets, lymphocytes), which has independent predictive value for various disease risks and long-term adverse survival outcomes compared to individual white blood cell subgroup counts (8). A study has shown a positive correlation between SII and neutrophil-to-lymphocyte ratio (NLR) with the prevalence of hypertension (9). Studies have revealed that elevated levels of the Systemic Inflammatory Response Index (SIRI) are significantly associated with stroke risk in elderly hypertensive patients (10), and that SIRI is also linked to osteoporosis and future fracture risk in these patients (11). Another study has demonstrated a close association between SII and cardiovascular and all-cause mortality in US adults (12). Subsequent research further indicates a significant correlation between higher SII (continuous or categorized variables) and increased risk of cardiovascular disease mortality, as well as a nonlinear relationship between all-cause mortality and cancer mortality (13).

A recent meta-analysis showed that SII may be a potential biomarker for the occurrence of CVD, with an increase in SII being associated with an increased risk of various subtypes of CVD (14). Higher SII is independently associated with a higher risk of myocardial infarction, stroke, death, and rehospitalization for heart failure after coronary artery intervention in patients with coronary heart disease (6). The severity of SII is positively correlated with stable CAD, with a predictive sensitivity and specificity both exceeding 85% (15). In the general population, SII is significantly associated with all-cause, cardiovascular, and cerebrovascular mortality (16). Several recent studies propose that the SII, calculated using neutrophils, platelets, and lymphocytes, serves as an innovative inflammatory marker linked to the onset of diverse cardio- and cerebrovascular diseases, such as isolated tricuspid valve surgery (17), coronary artery disease (CAD) (6, 15), congestive heart failure (18), acute ischemic stroke (19), and acute myocardial infarction (12). Similarly, research has also confirmed that the SII is an effective indicator for predicting hypertension and its complications (7, 9), as well as the risk of mortality from CVD (12, 13, 16, 20). It is widely recognized that there are significant gender differences in the clinical characteristics and determinants of hypertension, with evidence of differences in risk factors, disease awareness, treatment, control, and progression (21, 22). Over the years, the impact of gender on the incidence, presentation, and long-term outcomes of CVD has been a focus of research (23, 24). Several lifestyle and environmental factors influence blood pressure (BP) and the occurrence of CVD in a gender-specific manner. These factors encompass the immune system, the renin-angiotensin-aldosterone system, and the sympathetic nervous system (23). Recent research suggests variations in the impact of sex chromosomes and sex hormones on BP regulation, the distribution of cardiovascular risk factors, and the occurrence of complications among individuals with hypertension (25). Regulation of immune cells and cytokines in the bodies of hypertensive patients is one possible mechanism for gender differences (26, 27).

The above studies have laid the foundation for this study. However, the predictive value of SII for the risk of death in hypertensive patients has not been validated in different genders. Hence, our study utilizes a comprehensive and nationally representative sample to examine the association between SII and the likelihood of all-cause and cardiovascular mortality in hypertensive patients of different genders. We aim to contribute fresh perspectives for early prevention and more advanced management strategies.

## Methods

### Study population

NHANES is a complex sampling design population survey project aimed at collecting health and nutrition information from residents of the United States (US). All data was collected through structured household interviews, mobile center physical examinations, and laboratory tests. The survey procedure received ethical approval from the National Center for Health Statistics (NCHS), and written informed consent was obtained from all participants. As this study is a retrospective analysis and poses no risk of exposure to personally identifiable information, no additional ethical review or informed consent is required.

**Figure 1:**
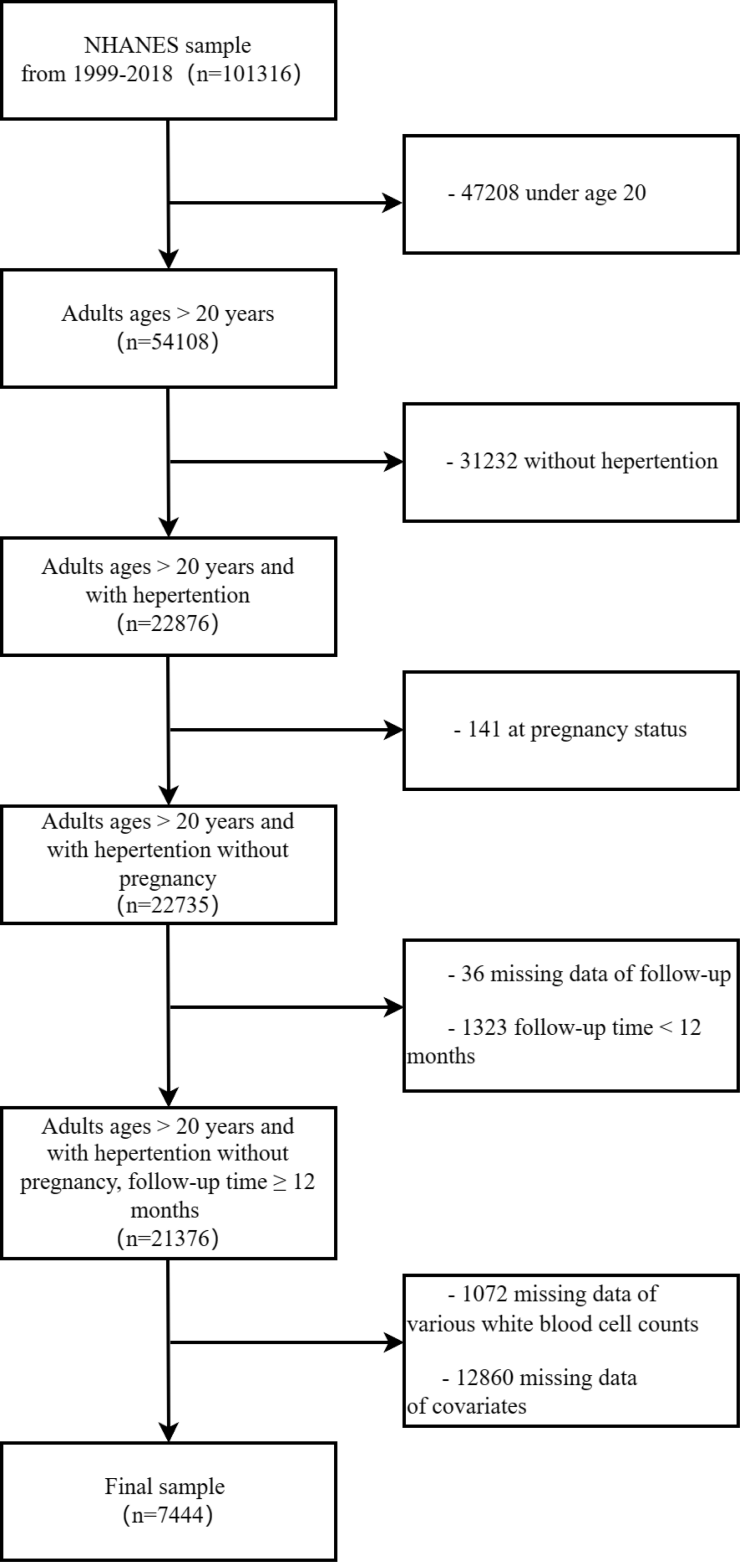
The diagram illustrating the inclusion and exclusion of participants in the present study.

From the data of NHANES 1999-2018, a total of 101,316 individuals were identified. Hypertension was defined in all survey cycles if any of the following three conditions were met: being informed of hypertension, taking medication for hypertension, or having an average systolic blood pressure ≥140 mmHg and/or average diastolic blood pressure ≥90 mmHg. In this study, the following criteria were utilized for selective exclusion: (1) age ≤ 20 years, (2) individuals not diagnosed with hypertension. (3) individuals with pregnant state, (4) individuals without survival status and follow-up time <12 months, with follow-up time available in mortality files, (5) individuals with missing WBC classification counts, (6) individuals with missing any other covariates. A final analysis was conducted on 7,444 subjects, as illustrated in the data selection process outlined in **Figure 1**. All data utilized in this study are accessible to the public without charge (https://wwwn.cdc.gov/nchs/nhanes/Default.aspx), and weighted methods were employed for subsequent analysis.

### Definition of SII

NHANES uses automated blood analysis equipment (Coulter^®^ DxH 800 Analyzer) to analyze various types of white blood cell counts, such as neutrophils, lymphocytes, and platelets, with the entire process supervised by trained medical staff. This study used a widely recognized formula to calculate the new target variable SII [SII = (neutrophils × platelets)/lymphocytes] (12, 13, 15).

### Main outcomes

The public-use mortality files from NHANES provide mortality data recorded in the survey registration up to December 31, 2019. These files enable the tracking of participant deaths and the identification of specific causes. The death codes adhere to the guidelines of the 10th edition of the International Classification of Diseases, Injuries, and Causes of Death (ICD-10). The variable MORTSTAT (final mortality status) is utilized to determine whether participants are alive or deceased. Mortality related to cardiovascular diseases (coded I00-I09, I11, I13, and I20-I51) was identified based on the ICD-10.

### Covariates

We incorporated various factors that could influence both SII and survival status (13). Key demographic details encompassed survey period, age, gender, race, education level, marital status, and health insurance. Smoking status was categorized as never, former, or current. Based on the United States Department of Agriculture’s criteria, alcohol consumption was classified into five groups—never, former, light, moderate, and heavy. Body mass index (BMI) was derived from examination center data and categorized as <25, 25-30, and ≥30. The poverty income ratio (PIR) measures the household income ratio to the federal poverty line, with lower values indicating higher levels of poverty. Laboratory indicators covered total cholesterol (TC), total triglycerides (TG), LDL, HDL, creatinine, blood urea nitrogen, and uric acid, all measured in mg/dl. The Tosoh Automated Glycohemoglobin Analyzer HLC-723G8 was designed for *in vitro* diagnostic quantification of % hemoglobin A1c (HbA1c) in whole blood samples. Self-reported physical activities—walking, cycling, home yard activities, muscle exercise, work activities, or entertainment—were marked as “yes” for physical activity. The estimated glomerular filtration rate (eGFR) for adults was determined using the CKD Epidemiology Collaboration (CKD-EPI) equation. Covariates included self-reported CVD (congestive heart failure, coronary heart disease, angina, or myocardial infarction), stroke (yes or no), and any type of cancer. Individuals were informed that meeting specific criteria—such as high blood sugar, HbA1c ≥ 6.5 mmol/L, fasting blood sugar ≥ 7.0 mmol/L, random blood sugar ≥ 11.1 mmol/L, oral glucose tolerance test (OGTT) ≥ 11.1 mmol/L, or current use of antidiabetic medications or insulin—defines the presence of diabetes.

### Statistical analysis

Following NHANES analysis and reporting guidelines, all analyses were weighted, considering the complex sampling design characteristics during the analysis process. The analyses were performed using R 4.2.2 software (the R Foundation for Statistical Computing, Vienna, Austria). Given the highly skewed concentrations of SII, the standardized data underwent a logarithmic transformation when considered as a continuous variable to approximate a normal distribution. Chi-square tests or t-tests were employed to evaluate participants’ baseline characteristics. Continuous variables were analyzed and reported as mean ± standard deviation (SD), while categorical variables were assessed by percentage with a 95% confidence interval (CI).

To identify the optimal SII cut-off point associated with the most significant impact on survival outcomes, the maximally selected rank statistics (MSRS) method was used to optimize the SII cut-off value (8, 28), which was applied using the “maxstat” package (version 0.7-25). The MSRS method does not require pre-setting a range of cut-off values and can select the optimal cut-off value from a wide range of possible values. The resulting cut-off values were then utilized to categorize participants into higher- and lower-SII groups. The relationship between SII (numerical variable) and survival is considered as part of the sensitivity analysis.

Survey-weighted Cox regression analysis was employed to assess the association between SII and all-cause, as well as cardiovascular mortality, in hypertensive patients of different genders. The robustness of the three subtypes for testing results was adjusted using the crude model. Model 1 included adjustments for age, sex, race, marital status, education, PIR, BMI, smoking, alcohol, health insurance, and physical activity. Model 2 accounted for additional adjustments, including eGFR, creatinine, urea nitrogen, uric acid, HbA1c, HDL, LDL, TC, and TG. Model 3 accounted for additional adjustments, including diabetes, CVD, stroke, and cancer.

The survival outcome probabilities were determined utilizing the Kaplan-Meier method and compared through the log-rank test. Additionally, the accuracy of SII in predicting survival outcomes for hypertensive patients of different genders at various time points was evaluated using the time-dependent receiver operating characteristic (ROC) curve analysis, implemented with the “timeROC” package (version 0.4). Interaction analysis of age, race, PIR, and physical activity with SII binary classification across different genders, and their association with mortality, was subsequently conducted. Throughout all analyses, statistical significance was set at a two-tailed *P*-value < 0.05.

## Results

### Characteristics of the study population

**Figure 2:**
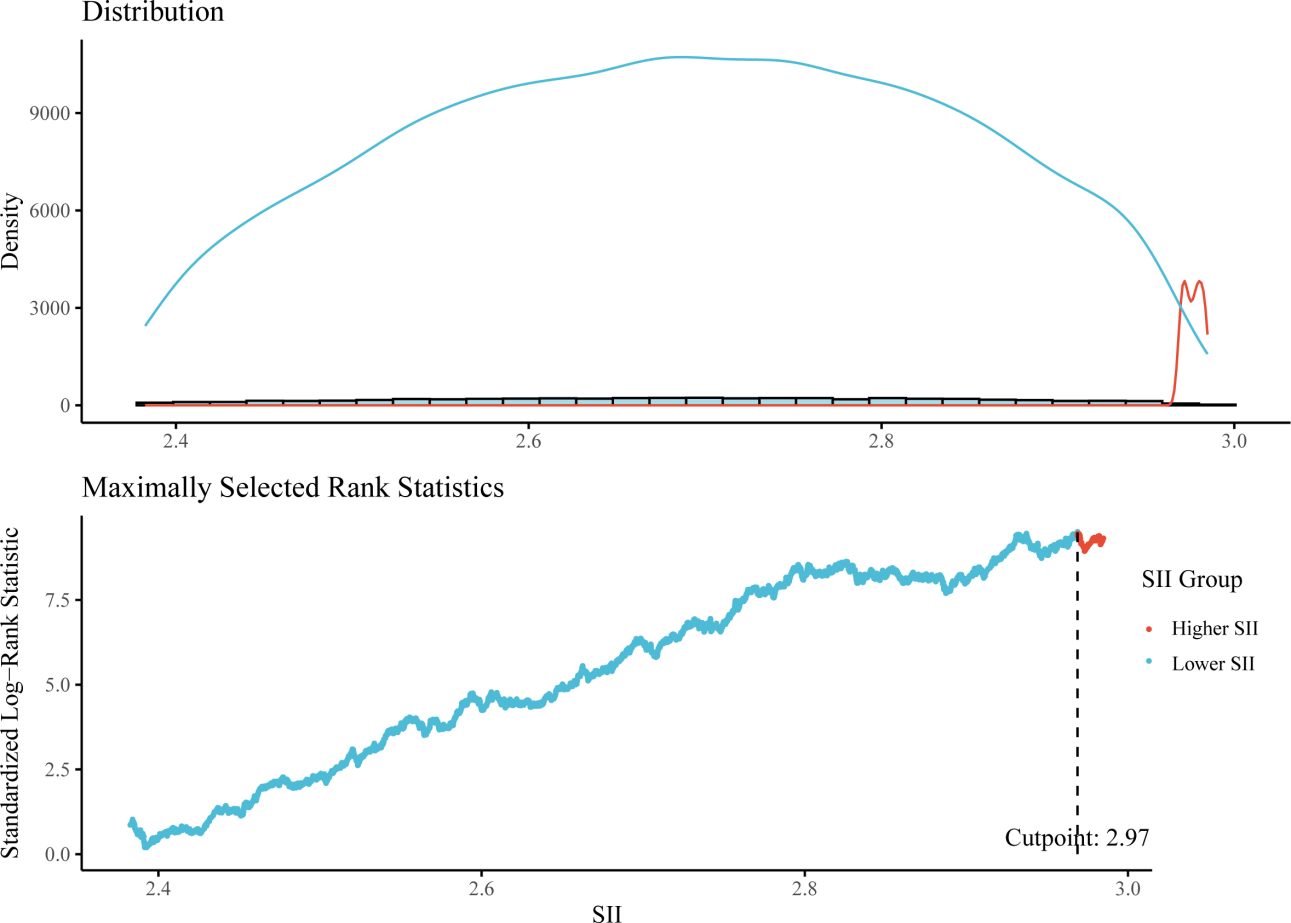
The cut-off point for lgSII calculated using maximally selected rank statistics.

This study included 7,444 hypertensive patients, representing 28,927,504 hypertensive patients in the US. The overall mean follow-up time was 111.43 months, with a total of 18.98% of participants deceased and cardiovascular mortality accounting for 5.31%. According to the MSRS method, using the optimal SII cut-off value (2.70) most significantly associated with survival, participants were divided into the higher SII group (SII > 2.70, n = 807) and the lower SII group (SII ≤ 2.70, n = 6637) (**Figure 2**). Compared to the lower SII group, participants in the higher SII group were older, more females, more individuals living alone, a higher percentage of smokers, more individuals in poverty, lower eGFR, LDL and TC levels, higher urea nitrogen level, more comorbidities including diabetes, stroke, cancer and CVD, less physical activity, and higher all-cause mortality. **Table 1** shows more detailed participant characteristics.

**Table 1:** Characteristic of the higher- and lower-SII groups among adults with hypertension.

Variable	Overall	Higher SII	Lower SII	*P*
	n = 7444	n = 807	n = 6637	
Year^b^				**0.01**
1999-2000	6.47 (5.19, 7.74)	6.99 (4.95, 9.04)	6.40 (5.13, 7.67)	
2001-2002	9.33 (8.15, 10.50)	9.61 (6.43, 12.80)	9.29 (8.18, 10.40)	
2003-2004	9.86 (7.83, 11.89)	12.58 (7.91, 17.25)	9.53 (7.74, 11.32)	
2005-2006	10.08 (8.56, 11.60)	14.77 (9.63, 19.92)	9.52 (8.25, 10.79)	
2007-2008	10.06 (8.26, 11.86)	11.41 (7.00, 15.81)	9.90 (8.32, 11.47)	
2009-2010	9.75 (7.80, 11.70)	8.77 (5.30, 12.25)	9.87 (7.98, 11.75)	
2011-2012	11.75 (9.45, 14.05)	10.34 (7.17, 13.51)	11.92 (9.75, 14.08)	
2013-2014	12.21 (10.74, 13.67)	11.72 (8.47, 14.97)	12.26 (10.90, 13.62)	
2015-2016	11.24 (9.62, 12.86)	7.36 (4.89, 9.83)	11.71 (10.06, 13.35)	
2017-2018	9.26 (7.77, 10.75)	6.44 (3.77, 9.10)	9.60 (8.06, 11.14)	
Sex^b^				**0.01**
Female	50.33 (47.48, 53.18)	56.41 (52.03, 60.79)	49.60 (48.23, 50.98)	
Male	49.67 (46.63, 52.70)	43.59 (39.21, 47.97)	50.40 (49.02, 51.77)	
Race/Ethnicity ^b^				**< 0.001**
Non-Hispanic White	73.51 (67.69, 79.32)	80.93 (77.80, 84.07)	72.62 (70.19, 75.04)	
Non-Hispanic Black	11.90 (10.64, 13.16)	7.35 (5.61, 9.08)	12.45 (10.89, 14.01)	
Mexican American	5.13 (4.33, 5.92)	4.93 (3.32, 6.54)	5.15 (4.25, 6.06)	
Other	9.46 (8.28, 10.64)	6.79 (4.80, 8.77)	9.78 (8.48, 11.08)	
Age^a^	56.70 ± 0.24	58.58 ± 0.75	56.47 ± 0.26	**0.01**
Marital status^b^				**< 0.001**
Married or partner	66.05 (61.87, 70.23)	57.88 (53.17, 62.60)	67.03 (65.39, 68.67)	
Alone	33.95 (31.78, 36.12)	42.12 (37.40, 46.83)	32.97 (31.33, 34.61)	
Education^b^				0.83
Less than high school	6.61 (5.91, 7.31)	6.29 (4.82, 7.75)	6.65 (5.94, 7.36)	
High school or equivalent	38.64 (35.67, 41.62)	39.59 (34.70, 44.49)	38.53 (36.60, 40.45)	
College or above	54.75 (51.40, 58.09)	54.12 (49.18, 59.05)	54.82 (52.72, 56.93)	
Health insurance^b^				0.34
No	10.97 (9.92, 12.01)	9.84 (7.60, 12.08)	11.10 (10.06, 12.15)	
Yes	89.03 (84.02, 94.04)	90.16 (87.92, 92.40)	88.90 (87.85, 89.94)	
Smoke^b^				**0.002**
Never	49.19 (46.62, 51.76)	43.23 (38.73, 47.72)	49.91 (48.25, 51.56)	
Former	32.33 (29.73, 34.94)	32.92 (28.46, 37.39)	32.26 (30.67, 33.85)	
Now	18.48 (16.75, 20.20)	23.85 (20.18, 27.52)	17.83 (16.53, 19.13)	
Alcohol^b^				0.68
Never	12.05 (10.79, 13.31)	10.53 (8.17, 12.88)	12.24 (11.03, 13.45)	
Former	19.00 (17.02, 20.99)	21.07 (17.26, 24.89)	18.76 (17.27, 20.24)	
Mild	38.89 (36.19, 41.59)	38.48 (33.93, 43.03)	38.94 (37.00, 40.89)	
Moderate	14.60 (13.15, 16.06)	14.30 (11.22, 17.37)	14.64 (13.37, 15.91)	
Heavy	15.45 (14.14, 16.75)	15.63 (12.04, 19.21)	15.42 (14.08, 16.76)	
BMI (kg/m^2^) ^b^				**0.05**
< 25	19.52 (18.00, 21.04)	22.98 (19.30, 26.65)	19.10 (17.91, 20.29)	
25-30	32.62 (30.50, 34.74)	28.92 (25.02, 32.82)	33.06 (31.74, 34.38)	
≥ 30	47.87 (44.84, 50.90)	48.10 (43.45, 52.75)	47.84 (46.34, 49.34)	
PIR^b^				**0.003**
≤ 1.0	12.22 (10.95, 13.48)	14.47 (11.56, 17.37)	11.95 (10.76, 13.14)	
1.0-3.0	37.63 (34.76, 40.50)	42.04 (37.67, 46.40)	37.10 (35.14, 39.05)	
> 3.0	50.16 (46.67, 53.64)	43.50 (38.82, 48.17)	50.95 (48.80, 53.11)	
eGFR^a^	85.33 ± 0.39	81.82 ± 1.03	85.76 ± 0.42	**< 0.001**
Creatinine (mg/dl) ^a^	0.93 ± 0.01	0.95 ± 0.02	0.93 ± 0.01	0.15
Urea nitrogen (mg/dl) ^a^	14.83 ± 0.10	15.40 ± 0.30	14.76 ± 0.11	**0.04**
Uric acid (mg/dl) ^a^	5.85 ± 0.02	5.87 ± 0.07	5.85 ± 0.02	0.77
HbA1c, %^a^	5.82 ± 0.02	5.83 ± 0.04	5.81 ± 0.02	0.73
HDL (mg/dl) ^a^	53.32 ± 0.31	52.47 ± 0.70	53.43 ± 0.33	0.21
LDL (mg/dl) ^a^	115.98 ± 0.59	110.59 ± 1.56	116.63 ± 0.61	**< 0.001**
TC (mg/dl) ^a^	196.63 ± 0.68	190.43 ± 1.83	197.38 ± 0.69	**< 0.001**
TG (mg/dl) ^a^	136.69 ± 1.35	136.90 ± 3.56	136.66 ± 1.41	0.95
Diabetes^b^				**0.01**
No	74.72 (70.39, 79.04)	69.84 (66.11, 73.57)	75.30 (73.68, 76.92)	
Yes	25.28 (23.26, 27.31)	30.16 (26.43, 33.89)	24.70 (23.08, 26.32)	
Stroke^b^				**0.04**
No	94.69 (89.59, 99.80)	92.95 (91.00, 94.91)	94.90 (94.20, 95.60)	
Yes	5.31 (4.57, 6.04)	7.05 (5.09, 9.00)	5.10 (4.40, 5.80)	
Cancer^b^				0.08
No	85.94 (81.17, 90.72)	83.34 (79.99, 86.69)	86.25 (85.16, 87.35)	
Yes	14.06 (12.78, 15.34)	16.66 (13.31, 20.01)	13.75 (12.65, 14.84)	
CVD^b^				**< 0.001**
No	86.36 (81.88, 90.84)	81.20 (78.00, 84.39)	86.98 (85.93, 88.03)	
Yes	13.64 (12.22, 15.06)	18.80 (15.61, 22.00)	13.02 (11.97, 14.07)	
Physical activity^b^				**< 0.001**
No	27.09 (24.90, 29.28)	35.90 (31.52, 40.27)	26.03 (24.42, 27.64)	
Yes	72.91 (68.79, 77.03)	64.10 (59.73, 68.48)	73.97 (72.36, 75.58)	
Status^b^				**< 0.001**
Assumed alive	81.02 (76.39, 85.64)	67.87 (63.87, 71.87)	82.60 (81.47, 83.72)	
Assumed deceased	18.98 (17.50, 20.47)	32.13 (28.13, 36.13)	17.40 (16.28, 18.53)	
Follow-up time (month) ^a^	111.43 ± 1.55	112.30 ± 3.43	111.33 ± 1.59	0.78

^a^reported as mean ± standard deviation (SD); ^b^reported as percentage with a 95% confidence interval (CI); SII, systemic immune-inflammatory index; BMI, body mass index; TC, total cholesterol; TG, total triglycerides; HbA1c, hemoglobin A1c; PIR, poverty income ratio; eGFR, estimated glomerular filtration rate; CVD, cardiovascular disease; Bold indicates *P* <0.05.

### Associations of the SII with all-cause and cardiovascular mortality

We used survey-weighted Cox regression analysis to examine the relationship between SII and the risk of all-cause and cardiovascular mortality in hypertension, as shown in **Table 2**. In the crude model, we found that SII values were associated with an increased risk of all-cause and cardiovascular mortality (all *P <*0.001), and this relationship remained consistent in models 1-3. Furthermore, when SII was divided into higher- and lower-SII groups, individuals in the higher SII group were more likely to experience all-cause and cardiovascular mortality (all *P <*0.001 in models 1-3).

**Table 2:** The associations between SII and mortality in individuals with hypertension.

Character	Crude model		Model 1		Model 2		Model 3	
	HR (95% CI)	*P*	HR (95% CI)	*P*	HR (95% CI)	*P*	HR (95% CI)	*P*
All-cause mortality								
SII	2.22 (1.59, 3.10)	**<0.001**	1.80 (1.39, 2.35)	**<0.001**	1.71 (1.30, 2.23)	**<0.001**	1.74 (1.32, 2.28)	**<0.001**
SII category								
Lower SII	ref		ref		ref		ref	
Higher SII	1.83 (1.53, 2.18)	**<0.001**	1.46 (1.25, 1.71)	**<0.001**	1.42 (1.21, 1.65)	**<0.001**	1.41 (1.20, 1.64)	**<0.001**
Cardiovascular mortality								
SII	2.15 (1.44, 3.21)	**<0.001**	2.20 (1.47, 3.30)	**<0.001**	2.12 (1.41, 3.20)	**<0.001**	2.32 (1.53, 3.52)	**<0.001**
SII category								
Lower SII	ref		ref		ref		ref	
Higher SII	1.63 (1.29, 2.06)	**<0.001**	1.53 (1.24, 1.88)	**<0.001**	1.38 (1.11, 1.72)	**0.004**	1.42 (1.12, 1.80)	**0.004**

SII, systemic immune-inflammatory index; BMI, body mass index; TC, total cholesterol; TG, total triglycerides; HbA1c, hemoglobin A1c; PIR, poverty income ratio; eGFR, estimated glomerular filtration rate; CVD, cardiovascular disease; HR, hazard ratios; CI, confidence interval; Bold indicates P <0.05.

Crudel model: adjusted for none; ref: reference level/category.

Model 1: adjusted for age, sex, race, marital status, education, PIR, BMI, smoke, alcohol, health insurance, physical activity.

Model 2: adjusted for race, marital status, education, PIR, BMI, smoke, alcohol, health insurance, physical activity, eGFR, creatinine,

urea nitrogen, uric acid, HbA1c, HDL, LDL, TC, TG.

Model 3: adjusted for race, marital status, education, PIR, BMI, smoke, alcohol, health insurance, physical activity, eGFR, creatinine.

urea nitrogen, uric acid, HbA1c, HDL, LDL, TC, TG, diabetes, CVD, stroke, cancer.

### Gender differences in the associations of the SII with all-cause and cardiovascular mortality

We used survey-weighted Cox regression analysis to examine the relationships between SII and the risk of all-cause and cardiovascular mortality in different genders, as shown in **Table 3**. In the crude model, when SII was considered as a numerical and binary variable (higher- and lower-SII groups), we found that high levels of SII were associated with an increased risk of all-cause and cardiovascular mortality in hypertensive patients (all *P <*0.001), regardless of gender. In models 1-3, we still found that high levels of SII were associated with an increased risk of all-cause mortality in female hypertensive patients (all *P <*0.001). We only found an association between higher SII and all-cause mortality in male hypertensive patients in model 2 (*P <*0.001), and this relationship disappeared after adjusting for all covariates (*P* = 0.05 in model 3). In model 3, whether SII was considered as a numerical or binary variable (higher- and lower- SII group), higher SII was associated with an increased risk of cardiovascular mortality in female hypertensive patients (all *P <*0.05), but this relationship did not exist in male hypertensive patients.

**Table 3:** Gender differences in the associations between SII and mortality among hypertension by Cox regression analysis.

Character	Crude model		Model 1		Model 2		Model 3	
	HR (95% CI)	*P*	HR (95% CI)	*P*	HR (95% CI)	*P*	HR (95% CI)	*P*
All-cause mortality								
Female								
SII	2.19 (1.35, 3.56)	**0.002**	2.42 (1.66, 3.54)	**<0.001**	2.26 (1.54, 3.31)	**<0.001**	2.27 (1.56, 3.30)	**<0.001**
SII category								
Lower SII	ref		ref		ref		ref	
Higher SII	1.83 (1.53, 2.18)	**<0.001**	1.46 (1.25, 1.71)	**<0.001**	1.42 (1.21, 1.65)	**<0.001**	1.41 (1.20, 1.64)	**<0.001**
Male								
SII	2.29 (1.44, 3.65)	**<0.001**	1.32 (0.90, 1.93)	0.16	1.21 (0.84, 1.74)	0.31	1.22 (0.84, 1.77)	0.30
SII category								
Lower SII	ref		ref		ref		ref	
Higher SII	1.97 (1.59, 2.43)	**<0.001**	1.33 (1.05, 1.69)	**0.02**	1.26 (1.00, 1.58)	0.05	1.26 (1.00, 1.58)	0.05
Cardiovascular mortality								
Female								
SII	2.58 (1.31, 5.10)	**0.01**	2.96 (1.34, 6.57)	**0.01**	2.62 (1.19, 5.77)	**0.02**	3.49 (1.63, 7.47)	**0.001**
SII category								
Lower SII	ref		ref		ref		ref	
Higher SII	1.64 (1.17, 2.29)	**0.004**	1.67 (1.18, 2.35)	**0.003**	1.41 (0.96, 2.07)	0.08	1.56 (1.08, 2.24)	**0.02**
Male								
SII	1.77 (1.07, 2.93)	**0.03**	1.69 (0.96, 2.97)	0.07	1.69 (0.96, 2.98)	0.07	1.64 (0.92, 2.92)	0.09
SII category								
Lower SII	ref		ref		ref		ref	
Higher SII	1.60 (1.18, 2.16)	**0.002**	1.33 (0.98, 1.79)	0.06	1.32 (0.94, 1.84)	0.11	1.28 (0.89, 1.82)	0.18

SII, systemic immune-inflammatory index; BMI, body mass index; TC, total cholesterol; TG, total triglycerides; HbA1c, hemoglobin A1c; PIR, poverty income ratio; eGFR, estimated glomerular filtration rate; CVD, cardiovascular disease; HR, hazard ratios; CI, confidence interval; Bold indicates P <0.05; ref, reference level/category.

Crude model: adjusted for none.

Model 1: adjusted for age, sex, race, marital status, education, PIR, BMI, smoke, alcohol, health insurance, physical activity.

Model 2: adjusted for race, marital status, education, PIR, BMI, smoke, alcohol, health insurance, physical activity, eGFR, creatinine,

urea nitrogen, uric acid, HbA1c, HDL, LDL, TC, TG.

Model 3: adjusted for race, marital status, education, PIR, BMI, smoke, alcohol, health insurance, physical activity, eGFR, creatinine.

urea nitrogen, uric acid, HbA1c, HDL, LDL, TC, TG, diabetes, CVD, stroke, cancer.

**Figure 3:**
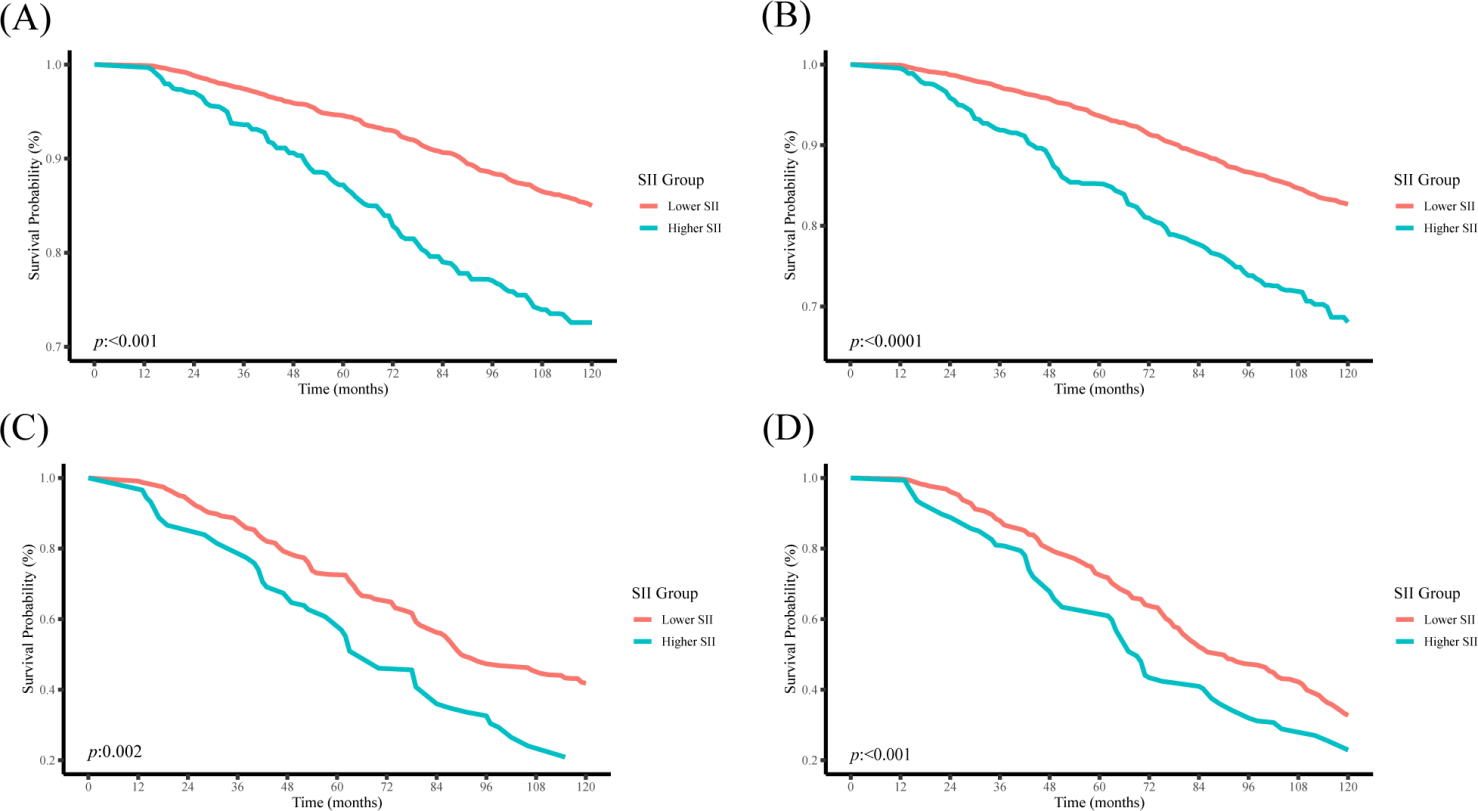
Kaplan-Meier curves depict the survival rate in different genders among the two groups. **(A)** All-cause mortality (female), **(B)** All-cause mortality (male), **(C)** cardiovascular mortality (female), **(D)** cardiovascular mortality (male).

In **Figure 3**, irrespective of gender, there were notable distinctions in Kaplan-Meier survival rates for both all-cause mortality and cardiovascular mortality between the higher- and lower-SII groups (all *P* < 0.05), with a lower survival rate observed in the higher SII group. Kaplan-Meier curves revealed that over time, the survival probability in the high SII group is significantly lower than that in the low SII group.

**Figure 4:**
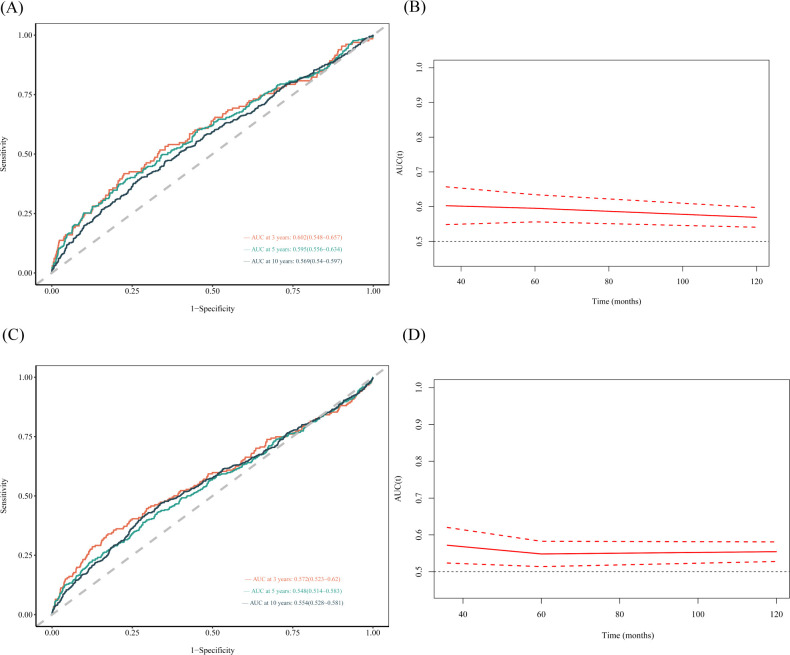
Time-dependent ROC curves and time-dependent AUC values of the SII for predicting all-cause mortality in different gender. **(A, B)** (female), **(C, D)** (male).

**Figure 5:**
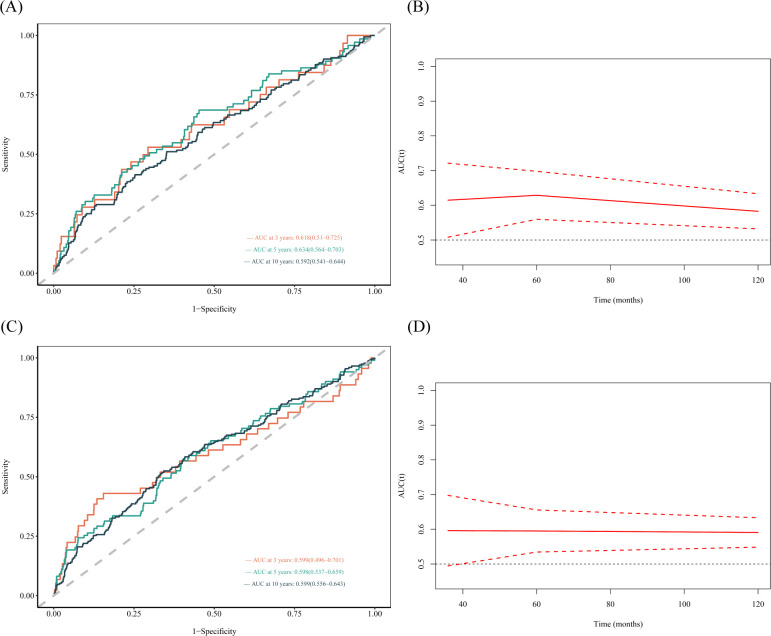
Time-dependent ROC curves and time-dependent AUC values of the SII for predicting cardiovascular mortality in different gender. **(A, B)** (female), **(C, D)** (male).

The results showed that the area under the curve (AUC) of the SII were 0.602, 0.595, and 0.569 for 3-, 5- and 10-year all-cause mortality, respectively, in females (**Figures 4A, B**). The AUCs of the SII were 0.572, 0.548, and 0.554 for 3-, 5- and 10-year all-cause mortality, respectively in males (**Figures 4C, D**). In **Figures 5A, B**, the results showed that the AUCs of the SII was 0.618, 0.634, and 0.592 for 3-, 5- and 10-year cardiovascular mortality, respectively, in females. The AUCs of the SII were 0.599, 0.598, and 0.599 for 3-, 5- and 10-year cardiovascular mortality, respectively, in males (**Figures 5C, D**). The time-dependent ROC curves demonstrated the predictive ability of SII at various time points, with slightly stronger predictive capacity observed in females compared to males. Higher area under the curve (AUC) values indicate that SII has greater accuracy in predicting mortality rates.

Additionally, we found that high levels of SII interact with PIR and physical activity to affect the mortality rate in the male population (*P* for interaction <0.05), while this relationship does not exist in the female population. High levels of SII also interact with race to affect the cardiovascular mortality rate in the female population (*P* for interaction <0.05), while this relationship does not exist in the male population (**Table 4**).

**Table 4:** Interaction analysis of the Age/Race/PIR/physical activity with SII among different gender.

Character	Lower SII	Higher SII^#a^	*P*	*P* for interaction	Higher SII^*a^	*P*	*P* for interaction
Female							
Age				0.45			0.59
<60	ref	1.57 (0.88, 2.80)	0.13		3.01 (1.22, 7.44)	0.02	
≥60	ref	1.97 (1.55, 2.50)	<0.001		1.59 (1.13, 2.24)	0.01	
Race/Ethnicity				0.96			**0.04**
Non-Hispanic White	ref	1.70 (1.24, 2.32)	<0.001		1.61 (1.05, 2.47)	0.03	
Non-Hispanic Black	ref	1.91 (1.10, 3.33)	0.02		4.28 (2.36, 7.75)	<0.0001	
Mexican American	ref	1.73 (0.85, 3.50)	0.13		1.47 (0.64, 3.36)	0.36	
Other	ref	1.67 (0.74, 3.77)	0.22		1.20 (0.34, 4.29)	0.78	
PIR	ref	1.06 (0.923, 1.22)	0.40	0.41	1.06 (0.92, 1.22)	0.40	0.41
Physical activity				0.37			0.53
no	ref	1.52 (1.08, 2.13)	0.02		1.82 (1.25, 2.66)	0.00	
Yes	ref	1.87 (1.28, 2.73)	0.00		1.65 (0.98, 2.76)	0.06	
Male							
age				1.00			0.33
<60	ref	1.69 (0.98, 2.93)	0.06		2.10 (0.94, 4.67)	0.07	
≥60	ref	1.70 (1.34, 2.15)	<0.001		1.41 (0.96, 2.08)	0.08	
Race/Ethnicity				0.84			0.18
Non-Hispanic White	ref	1.93 (1.53, 2.42)	<0.001		1.51 (1.08, 2.13)	0.02	
Non-Hispanic Black	ref	1.84 (0.10, 3.42)	0.05		1.77 (0.95, 3.29)	0.07	
Mexican American	ref	1.74 (0.77, 3.95)	0.18		2.77 (1.31, 5.86)	0.01	
Other	ref	2.80 (1.10, 7.11)	0.03		2.24 (0.81, 6.25)	0.12	
PIR	ref	1.24 (1.07, 1.42)	<0.001	**<0.001**	1.14 (0.90, 1.45)	0.27	0.28
Physical activity				0.98			**0.02**
No	ref	1.76 (1.30, 2.38)	<0.001		0.93 (0.60, 1.45)	0.75	
Yes	ref	1.78 (1.31, 2.43)	<0.001		2.04 (1.24, 3.37)	0.01	

SII, systemic immune-inflammatory index; PIR, poverty income ratio; ^#^All-cause mortality; ^*^cardiovascular mortality; ^a^reported as HR (95%CI); Bold indicates *P* <0.05; ref: reference level/category.

## Discussion

This large-scale population study indicates that there is a certain degree of gender difference in the relationship between SII and the survival prognosis of patients with hypertension. In the current study, high levels of SII are closely related to the high risk of all-cause and cardiovascular mortality in hypertensive patients. After adjusting for common risk factors, multi-variable analysis results show that this relationship is more pronounced in the female population. There is an interaction between higher SII and PIR, physical activity for the mortality rate in the male population, and an interaction between race and higher SII in cardiovascular mortality among the female population.

The systemic immune-inflammation index (SII) is an ideal parameter for comprehensive evaluation of the overall inflammatory status, with the advantages of wide application, simple measurement, low cost, and easy identification. SII integrates information on neutrophil, platelet, and lymphocyte counts, primarily reflecting inflammatory response, thrombosis, and immune reaction. Neutrophils, being the primary responders of the immune system, are frequently elevated in individuals with hypertension (29), and a decrease in neutrophils can alleviate hypertension induced by ischemia in rats (30). Moreover, neutrophils, to some extent, also contribute to multi-organ damage related to hypertension (31). Recent research on neutrophil extracellular traps (NETs) has highlighted a strong correlation between innate immunity, inflammation, oxidative stress, and cardiovascular diseases; elevated plasma levels of NETs are commonly observed in individuals with primary hypertension, correlating with accelerated development of atherosclerosis and thrombosis (32). Atherosclerosis is the leading cause of CVD (33), closely related to thrombosis, oxidative stress, endothelial cell injury, and immune inflammation (34). Furthermore, Mendelian randomization studies have confirmed a potential causal relationship between lymphocyte count and systolic and diastolic blood pressure (35). Previous research has focused on the involvement of different lymphocyte subgroups (including CD8^+^, Th1, Th2, Th17, and Treg cells) in the complex and multifaceted hypertension process (35, 36). Some scholars further emphasize that activated T cells regulate BP by decomposing reactive oxygen species and vascular endothelial cell factors, thereby altering the inflammatory environment in the blood vessels and kidneys (37). Recent research indicates the involvement of the gut microbiota in hypertension through a T lymphocyte-mediated inflammatory response (38). Similarly, higher platelet counts have been causally linked to an increased risk of hypertension (39). Overall, platelets play a role in the highly dynamic process of vascular diseases at different stages (40), and excessive activation or mutations of platelet receptors are crucial for the occurrence and development of cardiovascular diseases (41). Enhanced inflammatory responses, thrombosis, and immune system activation may exacerbate the risk of hypertension through various mechanisms, leading to higher mortality rates.

The results of previous studies on gender differences in hypertension are not entirely consistent, and these differences are reflected in aspects such as incidence, manifestations, long-term outcomes, and molecular mechanisms (21, 22, 42). For example, the incidence of hypertension in men is usually higher, but in women, BP rises sharply with age, leading to a higher incidence in the elderly (43). Women are more likely than men to experience inflammation and immune disorders, and they require lower BP to reduce the risk of cardiovascular events, possibly due to the protective effects of estrogen in lowering BP (44). The impact of gender on the manifestations and long-term outcomes of hypertension is complex; some studies have reported advantages for women in terms of cardiovascular risk, while others have suggested that complications and target organ damage may weaken these advantages (24). The mechanisms causing these differences include the renin-angiotensin-aldosterone system, sex hormones, and psychosocial factors (22). Some have suggested that women have a higher awareness of hypertension, but the treatment and control rates are similar between both sexes (21). Previous studies have identified variations in the prevalence, awareness, treatment, and control rates of hypertension between men and women in the US. Notably, women exhibit a lower likelihood of being aware of their hypertensive condition (45). The potential mechanisms of hypertension, including the renin-angiotensin system and sex hormones, are also influenced by gender (22). T cells are necessary for the development and maintenance of hypertension, and studies have shown gender differences in the regulation of T cells in hypertension, with females having more anti-inflammatory Treg cells and males having more pro-inflammatory T cells (46). These physiological differences may lead to differences in disease onset, susceptibility, prevalence, treatment response, and survival time (23). Despite these differences, the impact of gender on the effectiveness of antihypertensive drugs, cardiovascular disease incidence, and mortality rates is still unclear (24). Research consistently shows that there are gender differences in the relationship between CVD and mortality. Compared to women, men with hypertension have a 44% higher risk of developing CVD (47). However, women with hypertension are more likely to have cardiovascular risk factors such as central obesity, elevated creatinine, increased total cholesterol, and decreased high-density lipoprotein cholesterol (48). The mortality rate of heart disease in men accelerates at a relatively young age, but in women, the risk experiences a significant increase around the age of 60 years (49). The potential physiological differences between men and women can affect the pharmacokinetics and drug sensitivity of cardiovascular drugs. Therefore, many common cardiovascular drugs exhibit gender-specific therapeutic effects and adverse reactions (50). Young adult males much more frequently engage in most CVD-related risk behaviors and males have a higher level of CVD risk. Gender differences in CVD risk remain high even after adjustment for CVD lifestyles (51). These differences are influenced by various factors, including pathophysiology, risk factors, symptom presentation, and treatment outcomes (52). The role of sex hormones, particularly estrogen, in the pathophysiology of atherosclerosis and CVD is also highlighted (53). We found that the relationship between SII and the prognosis of hypertensive patients is more pronounced in women, which may be related to gender-specific immune responses and inflammatory pathways. Women may experience more pronounced inflammatory responses in hypertension due to hormonal regulation and social factors, which could exacerbate hypertension-related health risks.

Socioeconomic and psychosocial factors, as well as a potential gender bias in CVD management, may contribute to the higher CVD risk in women (54). The relationship between socioeconomic status (SES) and hypertension is controversial. A meta-analysis has shown that low SES is associated with an overall increased risk of hypertension, and this relationship is particularly evident in high-income countries (55). Neighbourhood deprivation appears to be a more robust predictor of hypertension in women. The prevalence of hypertension in women living in highly impoverished areas is significantly higher compared to both men within the same community and women residing in the least impoverished communities (56). Research has shown regional differences in the concentration of pro-inflammatory cytokines in the population with hypertension (57). In populations with low poverty income ratios, SII might increase due to prolonged socioeconomic stress and adverse living conditions, thereby exacerbating the risk of hypertension. This interaction may reflect the amplifying effect of socioeconomic disadvantages on inflammatory responses, suggesting that attention should be given to changes in inflammatory markers when addressing hypertension in low SES populations. Numerous studies have consistently demonstrated that physical activity is a protective factor for all-cause and cardiovascular mortality in hypertensive patients; for instance, there is an inverse dose-response pattern between physical activity and all-cause mortality across all BP levels (58, 59). Engaging in high levels of physical activity and maintaining a positive attitude towards it correlated with reduced BP levels (60). One of the critical mechanisms by which physical activity seems to benefit health is its ability to improve chronic low-grade inflammation throughout the body (61). SII may reveal the protective effect of physical activity on inflammation. In individuals with high SII levels, the benefits of physical activity might be more pronounced. This suggests that when developing hypertension intervention strategies, individual SII levels should be considered, and physical activity should be emphasized as a modifiable intervention for inflammation. Our study found that gender may play a significant role in the interactions between poverty, physical activity, and SII. The relationship between high hypertension risk in women in high-poverty environments and SII may be related to their physiological and social roles. Future research should further explore the mechanisms of these interactions to improve hypertension prevention and management strategies and enhance the effectiveness of clinical interventions.

It is important to acknowledge several limitations in this study. Firstly, as an observational study, causal relationships cannot be definitively established, even though we have made adjustments for potential confounding factors. Secondly, due to constraints within the database itself, multiple SII data points are scarce for thorough validation of this association. Thirdly, the absence of detailed information on hypertension subtypes and stages hinders exploration into potential variations in the relationship between SII and prognosis among patients with differing degrees or types of hypertension. Fourth, due to the lack of complete data on autoimmune diseases in the database, we were unable to explore the impact of other autoimmune diseases on the relationship between SII and mortality. Lastly, the findings of this study, drawn from a large sample population in the US, require further investigation to determine their applicability to other populations.

## Conclusion

The study revealed that higher levels of SII may be closely related to the high risk of all-cause and cardiovascular mortality in hypertensive patients, and this relationship is more significant and stable in the female group. High SII interacts with PIR, physical activity, and race to affect the mortality rate in different gender populations. More research is needed to discover the mechanisms underlying this gender difference.

## Data Availability

Publicly available datasets were analyzed in this study. This data can be found here: https://wwwn.cdc.gov/nchs/nhanes/Default.aspx.
